# Identifying Hendra Virus Diversity in Pteropid Bats

**DOI:** 10.1371/journal.pone.0025275

**Published:** 2011-09-28

**Authors:** Ina Smith, Alice Broos, Carol de Jong, Anne Zeddeman, Craig Smith, Greg Smith, Fred Moore, Jennifer Barr, Gary Crameri, Glenn Marsh, Mary Tachedjian, Meng Yu, Yu Hsin Kung, Lin-Fa Wang, Hume Field

**Affiliations:** 1 Australian Animal Health Laboratory, CSIRO Livestock Industries, East Geelong, Victoria, Australia; 2 Public Health Virology, Queensland Health Forensic and Scientific Services, Coopers Plains, Queensland, Australia; 3 Department of Employment, Economic Development and Innovation, Queensland Centre for Emerging Infectious Diseases, Biosecurity Queensland, Coopers Plains, Queensland, Australia; University of Hong Kong, Hong Kong

## Abstract

Hendra virus (HeV) causes a zoonotic disease with high mortality that is transmitted to humans from bats of the genus *Pteropus* (flying foxes) via an intermediary equine host. Factors promoting spillover from bats to horses are uncertain at this time, but plausibly encompass host and/or agent and/or environmental factors. There is a lack of HeV sequence information derived from the natural bat host, as previously sequences have only been obtained from horses or humans following spillover events. In order to obtain an insight into possible variants of HeV circulating in flying foxes, collection of urine was undertaken in multiple flying fox roosts in Queensland, Australia. HeV was found to be geographically widespread in flying foxes with a number of HeV variants circulating at the one time at multiple locations, while at times the same variant was found circulating at disparate locations. Sequence diversity within variants allowed differentiation on the basis of nucleotide changes, and hypervariable regions in the genome were identified that could be used to differentiate circulating variants. Further, during the study, HeV was isolated from the urine of flying foxes on four occasions from three different locations. The data indicates that spillover events do not correlate with particular HeV isolates, suggesting that host and/or environmental factors are the primary determinants of bat-horse spillover. Thus future spillover events are likely to occur, and there is an on-going need for effective risk management strategies for both human and animal health.

## Introduction

Hendra virus (HeV) belongs to the genus *Henipavirus* (family *Paramyxoviridae*, subfamily *Paramyxovirinae*), and is an emerging zoonotic virus [1], [2]. The virus is transmitted to humans via an intermediary equine host from bats of the genus *Pteropus* (Order *Chiroptera*, suborder, *Megachiroptera*, Family *Pteropodidae*) [3], [4], [5]. These bats are colloquially referred to as flying foxes.

The HeV virion is pleomorphic, measuring between 38 and 600 nm with a double-fringed envelope as viewed by negative contrast staining under the electron microscope [6]. The genome of HeV consists of a nonsegmented, single stranded, negative sense RNA of 18234 nucleotides that conforms to the “rule of six” for paramyxoviruses [7]. The genomic RNA forms a ribonucleoprotein core with the three viral proteins, the nucleoprotein (N), the phosphoprotein (P) and the large (L) protein which function in replication and transcription of the genome. The genomic order is 3′- N, P, matrix (M), fusion (F), glycoprotein (G), L-5′. The P gene also codes multiple non-structural proteins, the C, V, W and a putative SB protein [7].

HeV was first identified in 1994 following an outbreak in Hendra, a suburb of Brisbane, Queensland, Australia that resulted in the infection of 20 horses and two humans [8], [9]. There have been 31 identified spillovers of Hendra virus, resulting in a total 66 attributed equine cases and 7 human cases resulting in 4 human deaths (Table 1) [10]–[22]. In an unprecedented year for HeV activity, 17 spillovers resulting in 21 infections in horses have been identified between June and August 2011. The first infection in a dog was also diagnosed. Due to its wide host range, high mortality and lack of effective prevention or treatment modalities, HeV is classified in the highest biological safety category 4 (BSL4).

**Table 1:** Outbreaks of HeV in humans and horses.

Date	Location	Outcome	Sequence available
August 1994	Mackay, Queensland (QLD)	Death of two horses and one human [10], [11]	No
September 1994	Hendra, QLD	Death of 20 horses. Two humans infected, one death [8], [9]	Yes
January 1999	Cairns, QLD	Death of one horse [12]	No
October 2004	Gordonvale, near Cairns, QLD	Death of one horse. One human infected [13], [14]	No
December 2004	Townsville, QLD	Death of one horse [14]	No
June 2006	Peachester, QLD	Death of one horse [13]	Yes
November 2006	Near Murwillumbah, New South Wales (NSW)	Death of one horse [15]	Yes
June 2007	Peachester, QLD	Death of one horse [16]	Yes
July 2007	Clifton Beach, QLD	Death of one horse [17]	Yes
July 2008	Thornlands, Redlands, QLD	Death of five horses. Two humans infected, one death [18], [19]	Yes
July 2008	Proserpine, QLD	Death of four horses [17]	Yes
August 2009	Cawarral, QLD	Death of four horses. Death of one human [20], [21]	Yes
September 2009	Bowen, QLD	Death of two horses	Yes
May 2010	Tewantin, QLD	Death of one horse [22]	Yes
June 2011	Logan Reserve, QLD	Death of one horse	
June 2011	Kerry, QLD	Death of one horse	
June 2011	McLeans Ridges, NSW	Death of two horses	
July 2011	Mt Alford, QLD	Death of three horses, infection of one dog	
July, 2011	Utungun, NSW	Death of one horse	
July, 2011	Park Ridge, QLD	Death of one horse	
July, 2011	Kuranda, QLD	Death of one horse	
July, 2011	Hervey Bay, QLD	Death of one horse	
July, 2011	Corndale, NSW	Death of one horse	
July, 2011	Boondall, QLD	Death of one horse	
July, 2011	Chinchilla, QLD	Death of one horse	
July, 2011	Mullumbimby, NSW	Death of one horse	
August, 2011	Newrybar, NSW	Death of one horse	
August, 2011	Pimlico, NSW	Death of two horses	
August, 2011	Mullumbimby, NSW	Death of one horse	
August, 2011	Currumbin Valley, QLD	Death of one horse	
August, 2011	Tintenbar, QLD	Death of one horse	

Transmission from horses to humans has occurred following close contact with horses through performance of necropsies, during husbandry and veterinary procedures, and is thought to occur via respiratory droplets, cuts and abrasions, and mucous membranes. The mode of transmission from bats to horses has not been fully elucidated, but is thought to occur following ingestion of contaminated food or contact with contaminated surfaces [23]–[24].

To date, the increase in our knowledge of Hendra virus diversity has been incremental via sequence elaboration from sporadic equine spillover events that have effectively acted as sentinel surveillance (Table 1). This study sought to identify virus diversity in flying fox populations, and was an outcome of the three-year HeV surveillance study of free-living flying fox populations reported by Field *et al*. (2011) [25]. Our aim was to identify any correlation between particular variants and spillover to horses, and in turn provide more information for the development of risk management strategies and response preparedness. Equally importantly, the identification of variants circulating in the natural host ensure diagnostic assays remain current and sensitive to all HeV variants that might infect horses, ensuring that the likelihood of missed or delayed diagnosis is minimised.

## Materials and Methods

### Ethics

This study was approved by the Queensland Department of Employment, Economic Development and Innovation and the animal ethics permit number was SA 2008/10/270 and the approval for collection from native animals was approved by the Queensland Department of Environment and Natural Resources under permit number WISP05810609.

### Sample Collection

Details of the sample collection, handling and screening by PCR are presented in Field *et al.* 2011 [25]. In summary, colonies of flying foxes at roost were sampled by placing plastic sheeting under roosting sites shortly before flying foxes return to roost. Pooled urine samples were collected from the sheets approx one hour after returned to roost [26], [27]. Samples were held on wet ice in the field, transferred to the Biosecurity Sciences Laboratory (BSL) on wet ice (SEQ) or dry ice (FNQ), and stored at −70°C at the BSL. Prior to March 2009, samples were transferred to the Queensland Health Forensics and Scientific Services (QHFSS) laboratory for RNA extraction and screening by quantitative-PCR (qPCR); subsequently extraction and screening was done at BSL.

### Isolation of Hendra virus

Isolation from pooled urine samples was attempted at the CSIRO Australian Animal Health Laboratory at biological safety level 4 in Vero cells (ATCC CCL81), and primary *Pteropus alecto* cell cultures (kidney, foetal, lung) [28] grown in Dulbecco's Modified Eagle's Medium nutrient mixture F-12 HAM (Sigma) supplemented with foetal calf serum.

### Conventional PCR and sequencing

Amplification of viral RNA directly from urine was achieved by nested RT-PCR using high fidelity enzymes (Superscript III Platinum Taq (Invitrogen)) and virus specific primers. Areas initially targeted for sequencing were based on the positions of TaqMan assays that were in use in Australia.

First round cycling was performed on RNA to amplify a 2–3 kb product as follows: reverse transcription at 48°C for 30 mins then, initial denaturation at 94°C for 4 min, followed by 35 cycles of 94°C for 30 sec, 50°C for 30 sec, 68°C for 3 min then one cycle of 68°C for 7 min. Second round cycling was performed as above without the reverse transcription step using primer sets to amplify overlapping fragments. PCR products were electrophoresed on a 1% agarose gel and purified using the Qiagen gel extraction kit. Sequencing was performed using Big Dye Terminator 3.1 (Applied Biosystems) and reactions run on the Applied Biosystems 3130xl sequencer. All new sequence data has been deposited in GenBank (JN255800–JN255818).

### Full genomic sequencing of HeV isolates using Next Generation sequencing (454)

Following isolation of HeV, the tissue culture supernatant was clarified at 10,000 rpm for 20 min before ultracentrifugation through a 15% sucrose cushion to semi-purify of the virus. The virus pellet was resuspended in 350 µL RLT buffer and extracted using the RNeasy kit (Qiagen) according to manufacturer's instructions.

RNA was denatured at 65°C for 5 min in the presence of 1 µL of the cDNA primer (20 µM) 5′ GTTTCCCAGTAGGTCTCNNNNNNNN 3′ then placed on ice. The RNA was reverse transcribed at 50°C for 1 hour using Superscript III First Strand Synthesis system (Invitrogen) and 4 µL of 5× First Strand buffer, 1 µL 0.1 M DTT, 1 µL RNase OUT and 1 µl Superscript III. The sample was denatured at 100°C for 2 min then cooled on ice. Double strand synthesis was performed at 37°C for 30 min using 1 µL Klenow polymerase (Promega) following the addition of 2.5 µL of DNA polymerase buffer and 1 µL of the cDNA primer (20 µM). The enzyme was inactivated and then random PCR was performed on 2 µL of cDNA using GoTaqHotStart (Promega) and 4 µL of MID primer (20 µM) (MID1 5′acgaGTGCGTGTTTCCCAGTAGGTCTC3′, MID2 5′acgcTCGACAGTTTCCCAGTAGGTCTC 3′, MID3 5′agacGCACTCGTTTCCCAGTAGGTCTC 3′, MID4 5′agcaCTGTAGGTTTCCCAGTAGGTCTC 3′) to create template for 454 sequencing by amplification as follows: 1 cycle at 95°C for 5 min, 40 cycles at 94°C for 1 min, 50°C for 1 min, 72°C for 2 min and a final extension at 72°C for 7 min.

PCR products (10 µL) were electrophoresed on a 1% agarose gel in 1× Tris Acetate EDTA buffer to check that the amplification was successful. The remaining reaction was purified using the QIAquick spin kit (Qiagen). The purified products were processed for sequencing on the 454 Genome Sequencer FLX system (Roche) using titanium chemistry according to manufacturer's instructions for Rapid Library Preparation and emPCR Lib-L SV method manuals (454 Life Sciences, Branford, CT, USA). Any gaps in the sequence alignment, or any required confirmation of the sequence, were performed using conventional sequencing.

### Phylogenetic analysis

Data from the 454 Genome Sequencer (Roche) was analysed using the CLC Genomic Workbench v4.5.1 (CLC inc, Aarhus, Denmark) and CloneManager Professional v9.11 (Scientific and Educational Software, NC, USA). Alignments for Sanger sequencing were performed using SeqMan Pro v9.0.4 (DNASTAR). Phylogenetic analyses were undertaken using the MEGA 5 software package using neighbour-joining bootstrap analysis [29].

## Results

### Isolation of HeV from bat urine

Virus isolation was attempted from 30 samples positive by q-PCR, collected between Dec 2008 and March 2010 from multiple locations and species (Field et al 2011). Virus was isolated from 4 samples (Table 2). Although HeV has been isolated from foetal tissue and the reproductive tract of *Pteropus poliocephalus* and *Pteropus alecto* [4], the isolation of virus (on four occasions) in this study from pooled urine samples collected under-roost represents the first isolation of HeV from bat urine. Isolations from bat urine were equally successful in Vero cells and primary bat cell lines with no increased isolation in the primary bat cell lines. Interestingly, there was no correlation between a low Ct value (high quantity of RNA) in the TaqMan assay and the successful isolation of HeV. Similar findings have previously been observed when isolation attempts have been performed on human and horse samples [I. Smith, unpublished results]. This lack of isolation may be suggestive of non-viable virus being released during infection.

**Table 2:** Details of the q-PCR HeV positive urine samples from which HeV was isolated.

Location collected*	Species present	Date Collected
Cedar Grove, SEQ	*P. alecto* and *P. poliocephalus*	5 Aug 09
Yeppoon, CQ	*P. alecto*	13 Aug 09
Yeppoon, CQ	*P. alecto*	15 Aug 09
Tolga Scrub, FNQ	*P. conspicillatus*	24 Aug 09

*SEQ = South East Queensland, CQ = Central Queensland, FNQ = Far North Queensland.

### Sequencing at the sites of the TaqMan assays

Sequencing was undertaken on 11 samples. Initial sequencing of the HeV RNA centred around the locations of the HeV TaqMan assay primers and probes in the M gene and N gene [30], [31] in order to identify variability that could lead to a false negative diagnostic PCR result. There were two changes present, at nucleotide 1561 for the majority of the HeV variants and at nucleotide 1673 in the Peachester variant from 2007. These change/s were likely responsible for the failure of the N gene TaqMan assay to detect the Peachester 2006 and 2007 variants in naturally infected horses. There was one change at nucleotide 5815 in the area of the genome targeted by the reverse primer used in the M gene TaqMan in the bat isolates from Yeppoon, Cedar Grove and Tolga Scrub and in the variant isolated from a horse in Bowen. However, this change did not prevent the M gene TaqMan detecting the virus on these occasions.

### Whole genome sequence of four bat isolates

Next generation sequencing of the HeV isolates HeV/Australia/Bat/2009/Cedar Grove 11c, HeV/Australia/Bat/2009/Tolga Scrub 30g, HeV/Australia/Bat/2009/Yeppoon 4a and HeV/Australia/Bat/2009/Yeppoon 47a using the 454 Genome Sequencer (Roche), was undertaken to obtain sequence for the whole genome. The final trimmed data ranged from 2.3 MB to 13.3 MB of bases with between 73.3% (4877 bp missing) and 99.7% coverage (49 bp missing) of the HeV genome (Table 3). The quality of the material was proportional to the completeness of the genome.

**Table 3:** Coverage of the bat HeV isolates by 454 sequencing.

HeV isolate	Total bases (MB)	% bases HeV	% sequence coverage of the HeV genome
HeV/Australia/Bat/2009/Cedar Grove 11c	8.99	58.2%	99.6% (68 bp missing)
HeV/Australia/Bat/2009/Tolga Scrub 30g	2.3	9.5%	73.3% (4877 bp missing)
HeV/Australia/Bat/2009/Yeppoon 4a	13.3	46%	99.6% (69 bp missing)
HeV/Australia/Bat/2009/Yeppoon 47a	10.3	45%	99.7% (49 bp missing)

### Phylogenetic analysis

Sequence from a total of 10 samples from this study (6 variants sequenced from PCR amplification, plus 4 isolates) was included in the phylogenetic analysis. A segment of the HeV genome from nucleotide 1500 to 2240 was selected for initial amplification and analysis for variant variation. This region encompassed the hypervariable carboxyl terminal of the nucleoprotein gene, spanning the intergenic region between the nucleoprotein and the phosphoprotein. There were 39 nucleotide positions in this region with changes when the 25 HeV variants fully or partly sequenced were compared to the HeV reference sequence GenBank NC_001906 (Hendra 1994).

**Figure 1:**
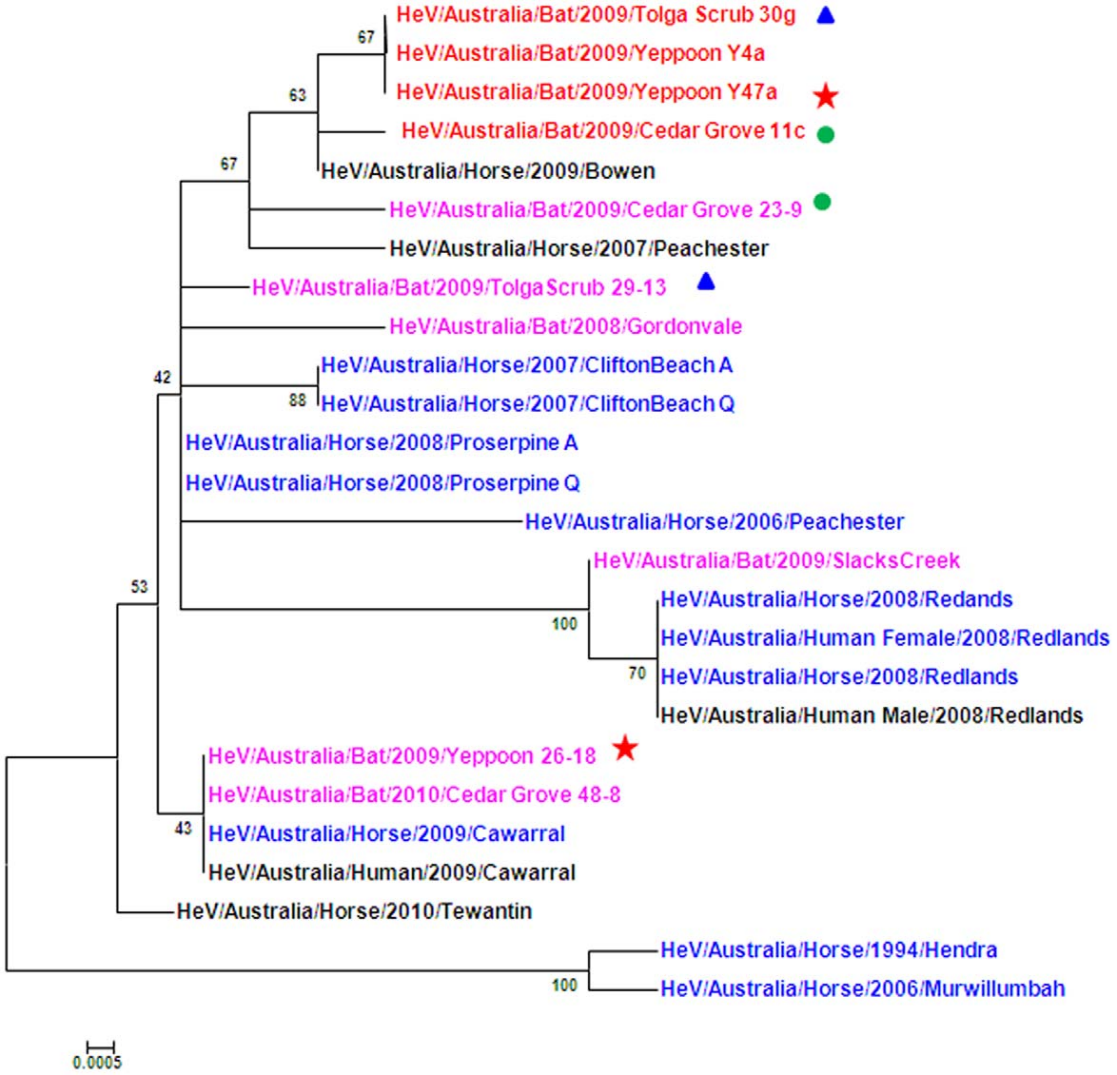
Phylogenetic tree (neighbour-joining, p distance) of the hypervariable region from nucleotide 1500 to 2240 (bat isolates of HeV are represented in red, horse isolates in blue. Bat variants sequenced from PCR amplification are indicated in pink and horse and human variants in black. Yeppoon collection 15/8/09, Cedar Grove collection 5/8/09, Tolga Scrub collection 24/8/09).

**Figure 2:**
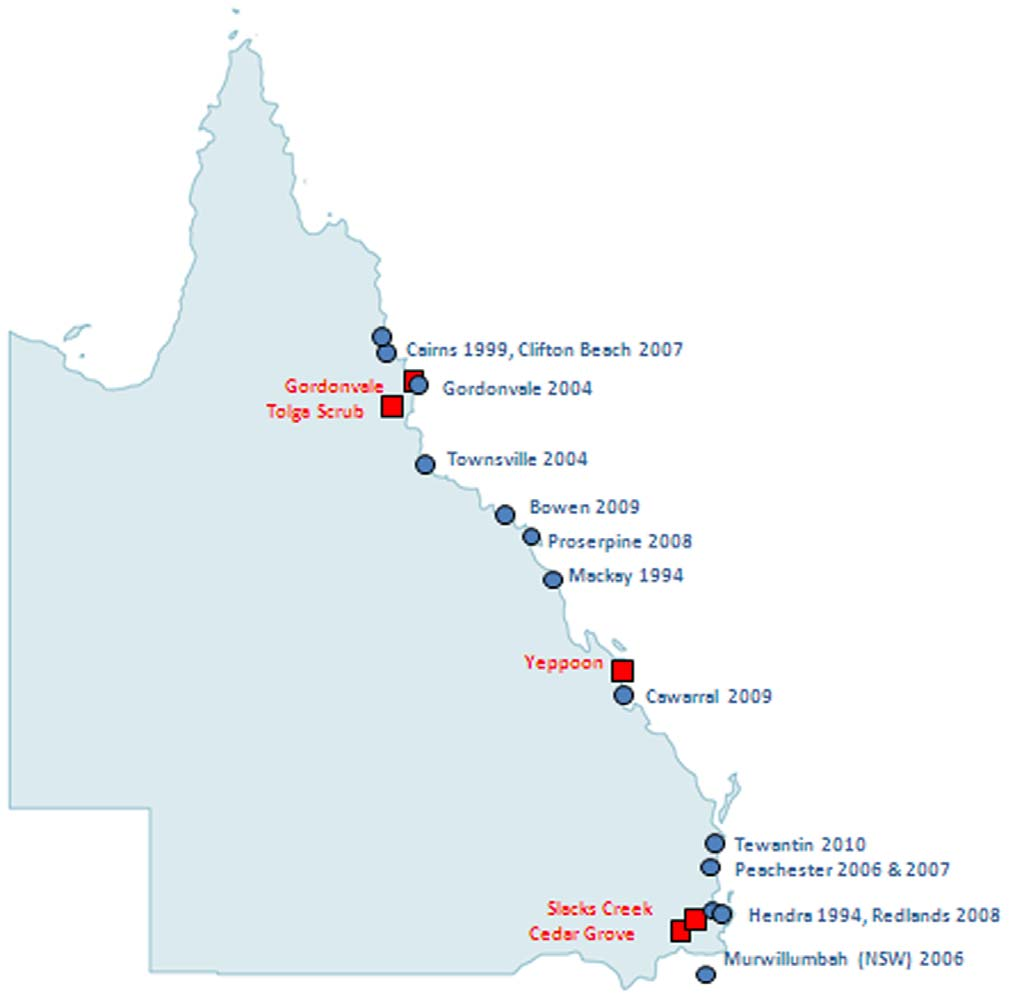
Map of Queensland with outbreaks in horses (blue circles) and sites of HeV detection from flying fox roosts (red squares).

Different variants of HeV were found to be present at the same time at the same location. For example, isolates HeV/Australia/Bat/2009/Yeppoon 47a and variant HeV/Australia/Bat/2009/Yeppoon 26-18 that were collected at the same location (Yeppoon) on the same date and were shown to be different (Figure 1, indicated by a star). Likewise, from Tolga Scrub, isolate HeV/Australia/Bat/2009/Tolga Scrub 30g and variant HeV/Australia/Bat/2009/Tolga Scrub 29-13 were collected on the same day but were different (Figure 1, indicated by a triangle) as was the isolate HeV/Australia/Bat/2009/Cedar Grove 11c and variant HeV/Australia/Bat/2009/Cedar Grove 23-9 from Cedar Grove (Figure 1, indicated by a circle). In addition, the same variants HeV/Australia/Bat/2009/Tolga Scrub 30g, HeV/Australia/Bat/2009/Yeppoon 4a and HeV/Australia/Bat/2009/Yeppoon 47a were found to be circulating in two different locations (Tolga Scrub and Yeppoon) which are 850 km apart (Figure 2).

**Figure 3:**
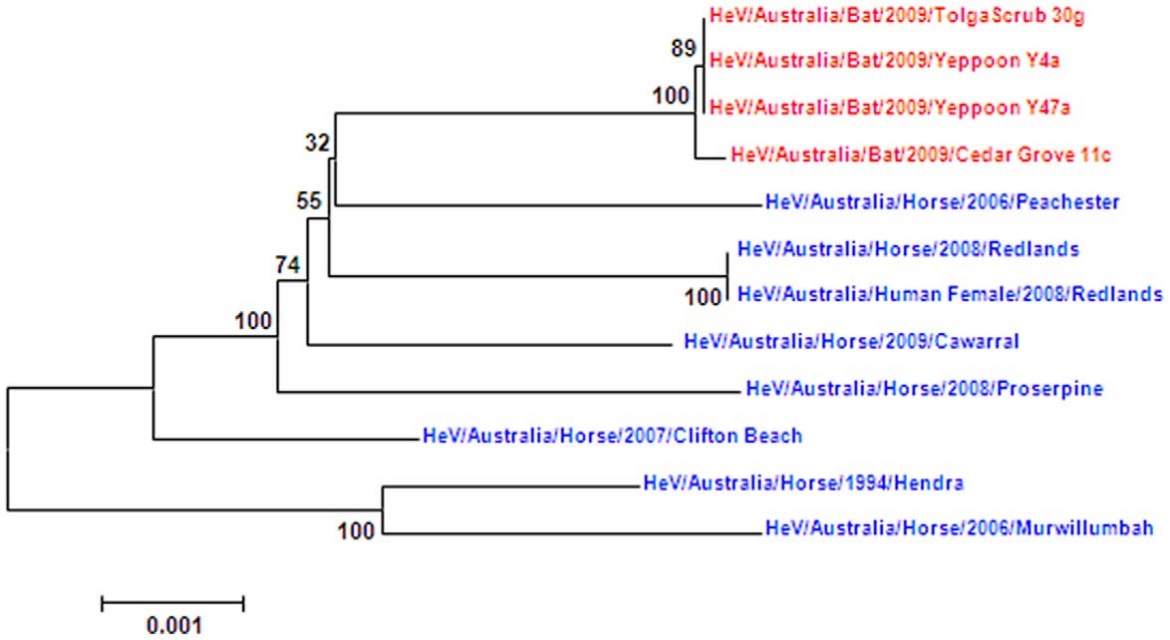
Phylogenetic tree of full length HeVgenome (bat isolates are represented in red, horse isolates are represented in blue).

Some geographical grouping was observed with the HeV/Australia/Bat/2009/Slacks Creek variant displaying greatest similarity with the outbreak variants HeV/Australia/Horse/2008/Redlands; and the HeV/Australia/Bat/2009/Yeppoon 26-18 variant showing similarity to HeV/Australia/Horse/2009/Cawarral. Comparison of the phylogenetic tree generated from the full length HeV sequences (Figure 3) revealed similar clustering to the 740 bp N-P region from nucleotide 1500 to 2240 (Figure 1). In both cases the newly isolated bat variants grouped together.

**Figure 4:**
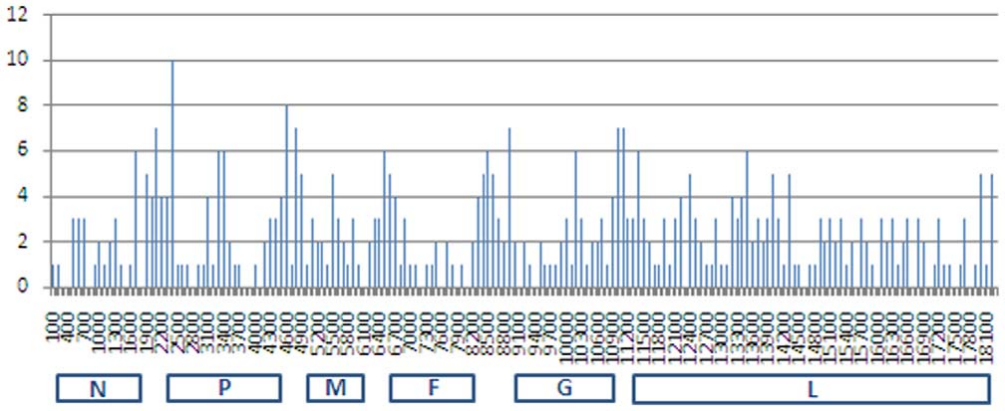
Variability map of the number of changes/100 nucleotides of HeV genome utilising the 12 full length sequences. Coding regions for the six major viral proteins are indicated beneath variability map.

In order to assess other regions in the HeV genome for their suitability for phylogenetic analysis, a plot of the number of changes over the length of the HeV genome was conducted (Figure 4). Regions of greatest variability were centred around each of the intergenic regions, although an area of variability was observed in the P gene between nucleotide 3000 and 3600 (Figure 4). When the 11 full length sequences of HeV variants were compared with the reference sequence, there were 155 positions in the intergenic regions and 305 positions in the coding regions of the genome where there were changes. This equates to 2.5 times more changes per length of sequence occurring in the intergenic regions than in the coding region when compared with the reference variant.

**Figure 5:**
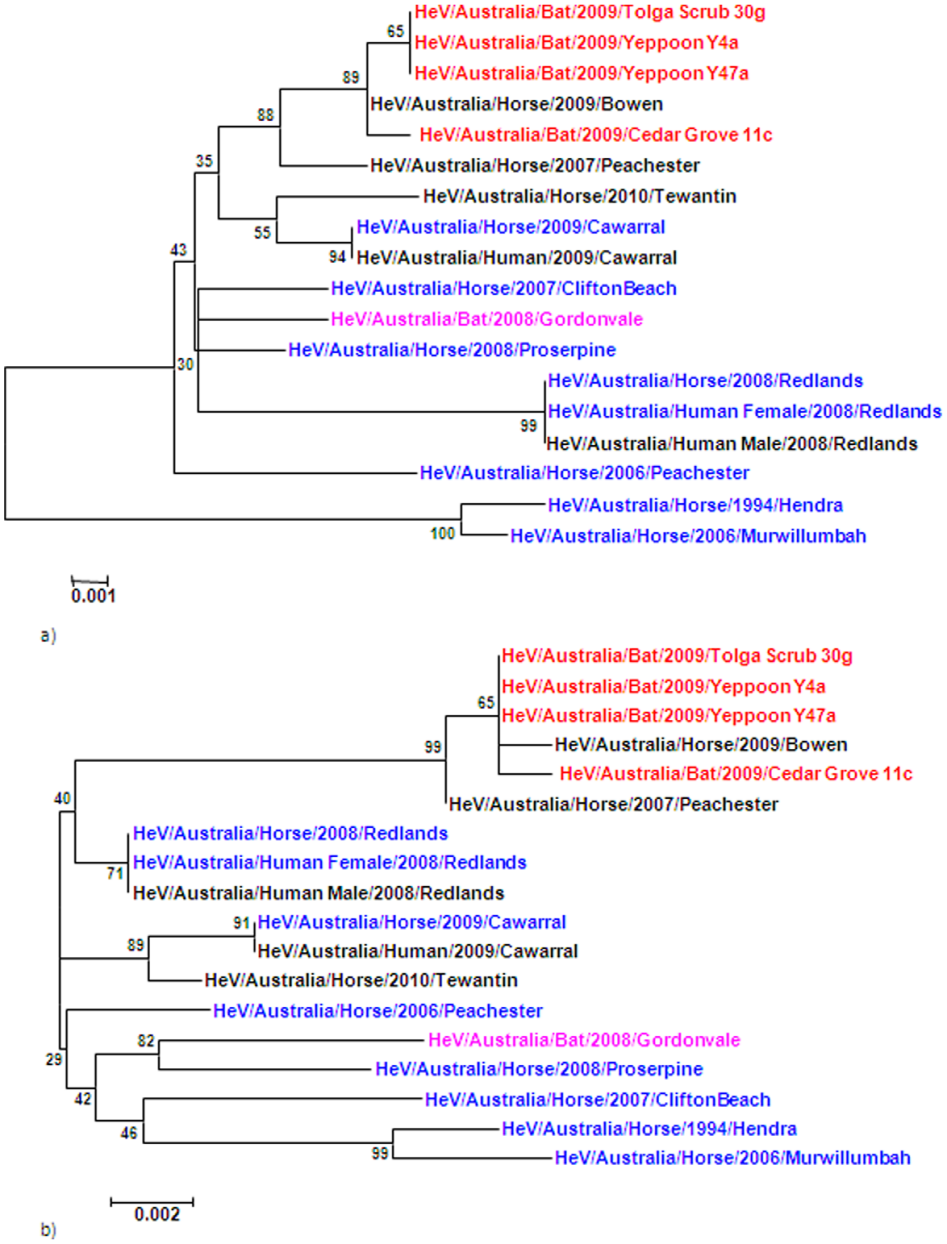
Comparison of the phylogenetic tree from two regions of the HeV genome a) nucleotides1561–2385 and b) nucleotides 4288–5065.

Based on the analysis, the region encompassing the N-P (nucleotide 1561–2385) and P-M (nucleotide 4288–5065) intergenic regions within the Hendra genome could be used to differentiate the HeV variants as these produced phylogenetic trees with similar clustering patterns to the full length genome with good differentiation (Figure 5).

## Discussion

The current study has expanded our knowledge of Hendra virus variant diversity and provided an insight into the HeV variants circulating within the flying fox populations.

Diversity of the HeV variants circulating within the migrating populations of flying foxes was observed, with multiple HeV variants circulating at the one time at multiple locations (Cedar Grove, Yeppoon, Tolga Scrub). In addition, the same variant was circulating at multiple locations as evidenced by the isolation of virus from both Yeppoon and Tolga Scrub, which are over 860 km apart by direct distance.

Analysis of the hypervariable region of the genome allowed for variants of HeV to be differentiated from one another by nucleotide changes, despite the overall genome displaying genetic stability as previously observed [32]. This genetic stability suggests that HeV is well adapted to its host, with coevolution resulting in minimal pressure to change over time. There were 2.5 times as many changes per length of sequence in the intergenic regions as there were in the coding regions when compared to the Hendra 1994 reference variant, suggesting that either changes in the intergenic regions are tolerated more than changes in coding regions, or that there is less selective pressure in the intergenic regions.

Similar clustering of isolates was observed regardless of the hypervariable region selected for analysis, although some regions gave better differentiation. Due to possibilities for variation in the genome, it is recommended that multiple TaqMan assays be used for the diagnosis of HeV. In addition, ongoing monitoring of HeV variability in bat populations is crucial for maintaining confidence in detection of variants.

Multiple variants circulating at the one time were observed previously in July 2008 when there were two concurrent outbreaks in horses over 930 km apart (direct distance) in the Redland suburb of Thornlands, and in Proserpine in Queensland. Concurrent outbreaks of HeV activity that spanned over 1400 kms was observed in August 2009, with the spillover of HeV at Cawarral (involving multiple fatal horse cases and one fatal human case) and the detections of HeV at Tolga Scrub, Yeppoon and Cedar Grove. Concurrent HeV activity was again detected in September 2009; in Bowen in a horse, and in Yeppoon and Cedar Grove in bats, a direct distance of over 600 km.

There were new introductions and a continual circulation of variants into a single flying fox population at Cedar Grove over the study period as the HeV variants (HeV/Australia/Bat/2009/Cedar Grove 11c, HeV/Australia/Bat/2009/Cedar Grove 23-9 and HeV/Australia/Bat/2010/Cedar Grove 48.8) are all different (Figure 1). This plausibly occurred with the migration of different species of flying fox into the roost, as that the typically predominantly *P. alecto* colony was periodically augmented by populations of *P. poliocephalus* and *P. scapulatus* during the study period (Field et al., 2011).

With such a mixture of variants circulating in flying fox populations throughout Queensland, it suggests that spillover events are not controlled by particular isolates, and that other factors that have led to the increase in outbreaks observed. These factors could include an improved awareness of HeV infection in horses, as well as an increase in the urbanisation of the flying fox populations leading to increased contact with horses and therefore humans [33]. Other factors in the flying fox population such as pregnancy and environmental stressors have also been suggested to increase HeV emergence [34].

Spillovers of HeV from flying foxes into horses are most likely due to the increased incidence of horses coming into contact with excretions from flying foxes when compared to humans. In addition, horses may be more susceptible to HeV infection as their innate immune response genes are genetically most closely related to flying foxes [35]. Intimate contact between horses and humans have been found to be required for infection, however, no direct transmission from bats to humans has been detected. The possibility of direct HeV infection from bats to humans cannot be ruled out. When spillovers occur from horses to humans the variation observed in HeV variants is minimal. There was 100% homology for the complete genome between isolates for a horse and the female human infected in the Redlands 2008 outbreak, whilst there was only one change observed in the intergenic region between the N and P gene in the fatal case human case from the same outbreak. There were only two changes detected between the variants infecting horse and human from the Cawarral 2009 outbreak.

This study has built on our understanding of the ecology and the frequency of HeV in flying fox colonies, although more research is needed to fully elucidate all mechanisms involved in the maintenance and transmission of HeV.
